# Effectiveness and Safety of Prostatic Artery Embolization for Patients with Prostate Cancer: A Systematic Review and Meta-Analysis

**DOI:** 10.1007/s00270-025-04107-6

**Published:** 2025-07-10

**Authors:** Vanesa Lucas-Cava, Francisco Miguel Sánchez-Margallo, Fei Sun

**Affiliations:** 1 Endoluminal Therapy and Diagnosis Unit, Jesús Usón Minimally Invasive Surgery Centre, Road N-521, Km 41.8, 10071 Cáceres, Spain; 2 Scientific Director, Jesús Usón Minimally Invasive Surgery Centre, Road N-521, Km 41.8, 10071 Cáceres, Spain

**Keywords:** Prostatic artery embolization, Prostatic artery chemoembolization, Prostate cancer, Meta-analysis

## Abstract

**Purpose:**

Prostatic artery embolization (PAE) has received attention to manage the urological symptoms in patients with prostate cancer (PCa). This meta-analysis evaluates its effectiveness and safety in patients with PCa.

**Material and Methods:**

A systematic review was performed by searching in PudMed and Web of Science databases for studies including either PAE or prostatic artery chemoembolization (PACE) in patients with PCa. Quantitative and qualitative analyses were performed. The primary outcomes were technical success, clinical success, oncological efficacy and adverse events (AEs). The secondary outcomes were International Prostate Symptoms Score (IPSS), quality of life (QoL), prostate volume (PV), and prostate-specific antigen (PSA).

**Results:**

Eleven single-arm studies with 151 participants were included. The pooled technical and clinical success rates were 95.53% (95%CI: 87.23, 99.95) and 90.31% (95%CI: 73.44, 99.85), respectively, whereas the oncological efficacy was 65.89% (95%CI: 32.18, 93.13). AEs showed low rates of minor 23.88% (95%CI: 8.88, 42.39) and major 0.6% (95%CI: 0.00, 3.67). Although, PAE tended to fewer AEs compared to PACE, 20.76% vs 31.03% for minor, and 1.01% vs 0.32% for major AEs, respectively. In addition, there was a statistically significant reduction in IPSS (− 10.24, 95%CI: − 14.60,− 5.89), QoL (− 2.28, 95%CI: − 3.25, − 1.32), PV (− 22.16, 95%CI: − 34.20, − 10.13), and PSA (− 7.32, 95%CI: − 12.34, − 2.29), with greater improvements after PACE. Overall, the studies showed a high risk of bias.

**Conclusion:**

This meta-analysis revealed that PAE and PACE are feasible, effective, and safe techniques to control the urological symptoms in patients with PCa, and even as adyuvant treatment for local therapies.

**Graphical Abstract:**

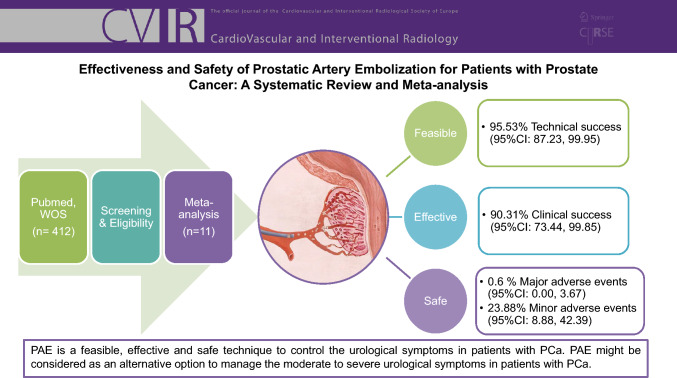

**Electronic supplementary material:**

The online version of this article (10.1007/s00270-025-04107-6) contains supplementary material, which is available to authorized users.

## Introduction

Prostate cancer (PCa) is the fourth most commonly diagnosed cancer in men, with an estimated 1.4 million cases worldwide in 2020, and the third cancer-related cause of death among men. In Europe, it is the most frequently diagnosed cancer in men with a prevalence of around 60% in patients over 75 years old [1].

At disease onset, most men with PCa are asymptomatic with a 5-year survival rate around 90% for localized PCa. Treatment in this stage varies from radical prostatectomy to radiation therapy, or even active surveillance in cases of low-risk disease. Despite these treatments, approximately 20 to 30% of men previously treated may experience recurrence and develop advanced stages of the disease, along with urological symptoms and poor quality of life [2].

The lower urinary tract symptoms (LUTS) in men related to an enlarged prostate can be managed with different minimally invasive treatment options, including prostatic artery embolization (PAE), which has demonstrated efficacy and safety to relieve moderate-to-severe LUTS related to benign prostatic hyperplasia (BPH) in patients [3, 4]. In addition, PAE has shown to stop the gross haematuria and remove of indwelling urinary catheter secondary to BPH in patients [5, 6]. The growing interest on PAE has extended its consideration beyond BPH, including potential application in the treatment of PCa in patients.

Currently, a few studies describe the role of PAE for the treatment of LUTS in the context of PCa [7–9], or even as adyuvant treatment with local therapies in patients with localized PCa [10, 11]. This systematic review and meta-analysis aims to summarize the current literature available on PAE and evaluate its effectiveness and safety in patients with PCa.

## Material and Methods

### Search Strategy and Study Selection

A systematic review was performed according to the Preferred Reporting Items for Systematic Reviews and Meta-analyses (PRISMA) guideline [12]. The review protocol was registered on PROSPERO (register number: CRD42024551978).

A systematic search was conducted in PubMed and Web of Science databases up to 1 April 2024 with English language restriction for studies including either PAE or prostatic artery chemoembolization (PACE) in patients with PCa. The full search strategy is presented in the supplementary information.

The eligible studies included patients with PCa underwent to either PAE or PACE. The exclusion criteria were: 1) a single-case report, review or abstract; 2) studies describing clinical outcomes after PAE together other treatments; 3) studies involving animals with PCa. An online systematic review tool (Parsifal) was used to manage the eligibility of the studies. After removing duplicated studies, two review authors (V.L.C. and F.S.) independently screened the titles and abstracts. For each potentially relevant study, the full-text article was assessed for eligibility. Discrepancies in article selection were resolved through consensus.

### Data Collection and Quality Assessment

Parsifal platform was used to manage the data extraction. The primary outcomes were technical success, clinical success, oncological efficacy, and adverse events. The clinical success was defined as the relief of LUTS measured by the decrease of International Prostate Symptoms Score (IPSS) of at least 30% from baseline data. The oncological efficacy was defined as tumour regression, meaning a decrease in size of prostate tumour and/or an absence of tumour progression from baseline, assessed by MRI. The severity of adverse events was classified according to Clavien–Dindo classification as minor for grades I–II, and major for grades III–IV complications related to procedure. The secondary outcomes were IPSS, quality of life (QoL), prostate volume (PV), and prostate-specific antigen (PSA). The data were extracted for the data synthesis.

The quality assessment was performed using the ROBINS-I tool for non-randomized trials [13]. Seven domains through which bias might be introduced were assessed for each study. Each individual bias item was assessed as “low risk”, “high risk”, or “some concerns”, giving an overall risk of bias judgement for each included study. In addition, *robvis* tool was used to create the figure of risk of bias assessment [14].

### Statistical Analysis

Data synthesis was performed using STATA, version 14.0 (StataCorp. 2015, Stata Statistical Software: Release 14; StataCorp LP, College Station, TX, USA). Heterogeneity was assessed by a visual inspection of forest plots and, subsequently, I^2^ statistic. A random effect model using the method of DerSimonian and Laird was applied for data pooling when I^2^ > 50%. A meta-analysis of proportion was conducting using the *metaprop* command for binomial data, whereas a pairwise meta-analysis was performed for continuous data. The pooled rates were expressed as effect size with 95% confidence intervals (CI) and continuous outcomes as weighted mean difference (WMD) with 95% CIs. The meta-analysis was carried out based on intervention subgroup. A meta-regression was conducted to determine if there was any factor which could affect to the results. The possibility of publication bias was analysed by Egger’s test. In addition, a sensitivity analysis was performed. The statistical significance was p value < 0.05.

## Results

### Study Characteristics

**Fig. 1:**
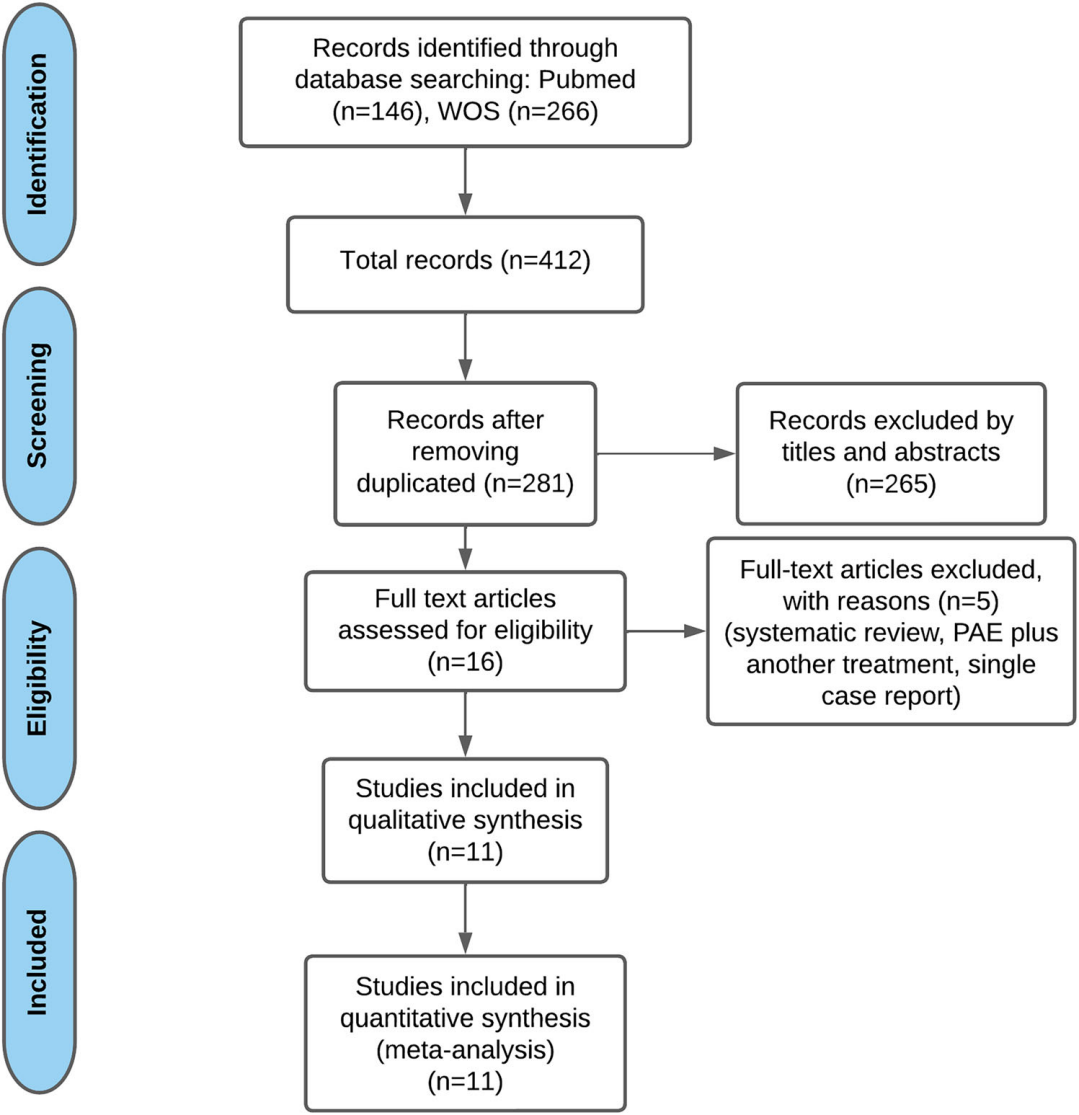
PRISMA flow chart of the search process

A flow chart of the search strategy and study selection is displayed in Fig. 1. A total of 412 articles were obtained from the two major databases. After removing 131 duplicated articles, 281 articles were screened based on titles and abstracts, of which 16 articles were retrieved for full-text review. Finally, 11 studies were selected for the meta-analysis, all of them single-arm trials. PAE was performed in 8 out of 11 studies [7–11, 15–17], whereas in the remaining three studies, the patients underwent PACE [18–20]. A total of 151 participants were enrolled with different stage of PCa (localized and advanced PCa), and symptoms, mainly LUTS, urinary retention with indwelling urinary catheter and/or haematuria in the most cases. The length of follow-up varies from 6 weeks to 24 months due to the different purpose of the studies. Details of the included studies and patients are shown in Table 1**.**

**Table 1:** Study characteristics of studies included in the meta-analysis

Study	Study	Patients	Clinical symptoms	Intervention	Follow-up (months)	Outcomes
Frandon et al. (2021)	Prospective single-centre study (NCT03407963)	Localized PCa (n = 10)	NA (under active surveillance)	PAE with 300–500 µm (Embospheres®)	1, 3, and 6	Technical success, oncological efficacy, procedure time, fluoroscopy duration, radiation dose, PSA, IPSS, QoL, EQ-5D-5L, IIEF-6, PV, tumour regression by histology and adverse events
Tapping et al. (2019)	Case series report	Advanced (n = 2) and localized PCa (n = 2)	Haematuria	PAE with 300–500 µm (Embospheres®)	3, 12 and 18	Technical success, clinical success, IPSS, EQ-5D-5L, IIEF-5, PV and adverse events
Haddad et al. (2022)	Retrospective single-centre study	Localized PCa (n = 13)	LUTS	PAE (calibrated spherical embolic agents)	2*	PSA, IPSS, PV and adverse events
Mordasini et al. (2018)	Bicentric prospective trial (NCT02917161)	Localized PCa (n = 12)	NA (prior to RARP)	PAE with 100 µm (Embozone®)	1.5*	Technical success, procedure time, fluoroscopy duration, radiation dose, embolic amount, PSA, IPSS, QoL, Qmax, PVR, IIEF-5, NIH-CPSI, prostate ischaemia, tumour regression by histology and adverse events
Malling et al. (2019)	Prospective single-centre study (NCT03104907)	Advanced PCa (n = 15)	LUTS and indwelling urinary catheter	PAE with 300–500 µm (Embospheres®)	1 and 6	Technical success, clinical success, procedure time, fluoroscopy duration, radiation dose, embolic amount, PSA, IPSS, QoL, Qmax, PVR, IIEF-5, PV and adverse events
Burkhardt et al. (2024)	Prospective single-centre study (NCT03457805)	Advanced PCa (n = 19)	LUTS	PAE with 250–400 µm (Embozone®)	3 and 12	PSA, IPSS, QoL, Qmax, PVR, PV, tumour response by MRI and adverse events
Pisco et al. (2018)	Prospective single-centre study	Localized PCa (n = 20)	Refusing standard therapy and LUTS	PACE with 100-300 µm (Embospheres®) and docetaxel	1, 12 and 18	Technical success, clinical success, procedure time, fluoroscopy duration, radiation dose, pain severity, PSA, IPSS, QoL, Qmax, PVR, IIEF-5, PV and adverse events
Chen et al. (2017)	Retrospective study	Advanced PCa (n = 9)	Refractory haematuria	PAE with PVA, Spongostan and coils	3	Technical success, clinical success, haemoglobin level, packed red blood cells and adverse events
Guan et al. (2022)	Case series report	Advanced PCa (n = 8)	LUTS, indwelling urinary catheter, and haematuria	PACE with 70-150 µm (Callispheres®) and epirubicin, 100-300 µm PVA	1 week, 1, 3, 6 and 12	Technical success, clinical success, prostatic artery origin, PSA, IPSS, QoL, IIEF-5, PV, and adverse events
Wang et al. (2023)	Prospective study (ChiCTR1900028819)	Advanced PCa (n = 32)	LUTS, haematuria and/or indwelling urinary catheter	PACE with 30-60 µm (Hepaspheres®), docetaxel and epirubicin,	1, 3 6, 12, 18 and 24	Technical success, clinical success, PSA, IPSS, QoL, IIEF-5, PV, prostate ischaemia, tumour response by MRI and adverse events
Parikh et al. (2021)	Retrospective single-centre study	Localized PCa (n = 21)	LUTS	PAE with 100-300 µm (n = 1) and 300–500 µm (n = 20) (Embospheres®)	1.5 and 3	Technical success, clinical success, procedure time, fluoroscopy duration, radiation dose, PSA, IPSS, QoL, PV, oncologic progression by MRI and adverse events

EQ-5D-5L, EuroQuality of life-5 Dimensions-5 Levels; IIEF, International Index of Erectile Function; IPSS, International Prostate Symptom Score; MRI, Magnetic Resonance Imaging; NIH-CPSI, Chronic Prostatitis Symptom Index; PVR, Post-Void Residual Volume; Qmax, Peak Flow Rate; QoL, Quality of Life; LUTS, Lower Urinary Tract Symptoms; PAE, Prostatic Artery Embolization; PACE, Prostatic Artery Chemoembolization, PCa, Prostate Cancer; PSA, Prostate-Specific Antigen; PV, Prostate Volume; RARP, Robotic-Assisted Radical Prostatectomy; PVA, Polyvinyl Alcohol Particles

^*^Follow-up after intervention, not including other treatments

### Evaluation of Quality Assessment and Publication Bias

**Fig. 2:**
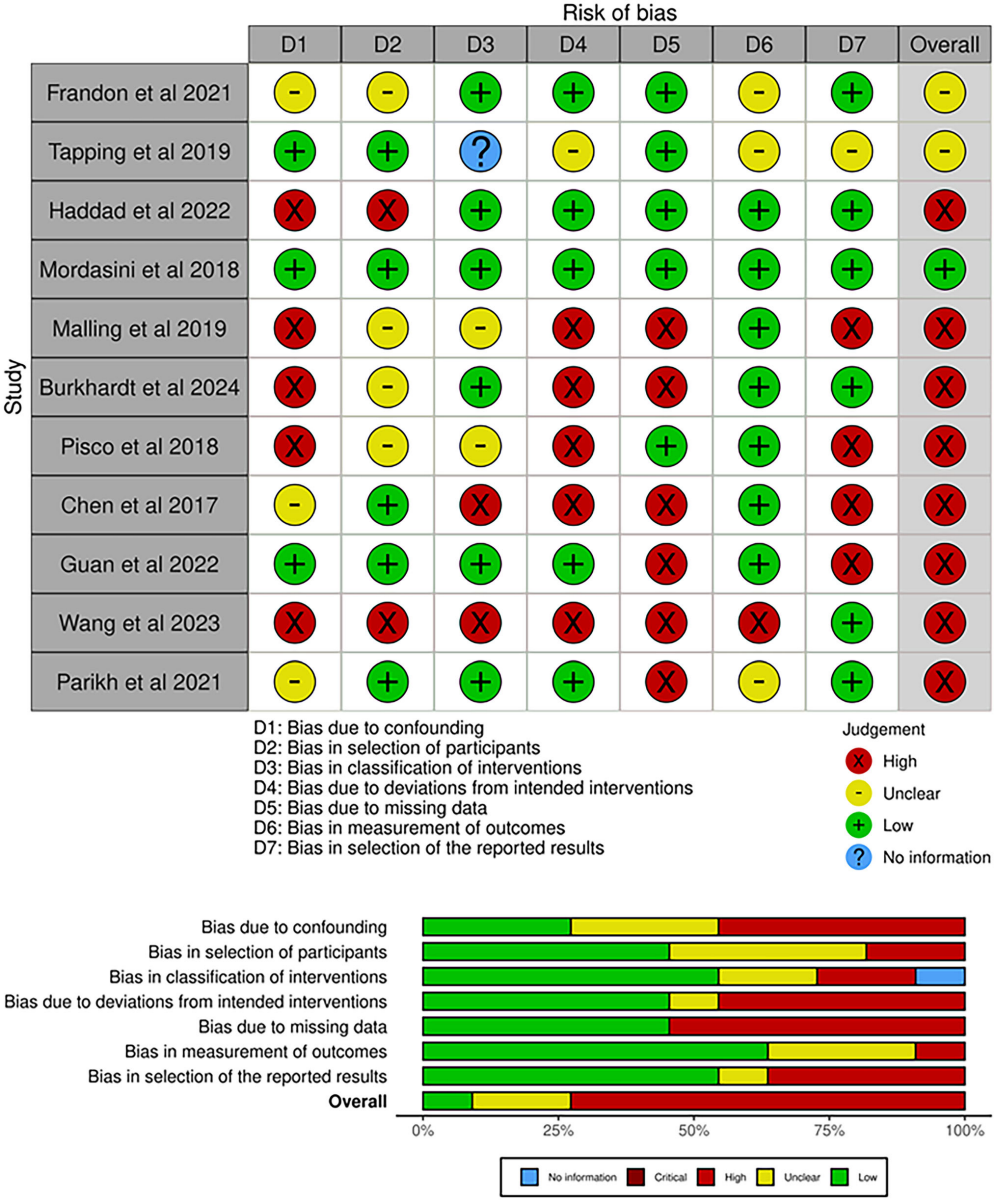
Risk of bias assessment using ROBINS-I tool

Overall, the included studies have shown to have high risk of bias except Mordasini’s study [11] which was considered to have a low risk of bias. All studies showed either high or some concerns risk of bias in some domains due to the complexity of PCa disease, where the severity of the symptoms and the disease make it difficult to follow the target trial. The risk of bias assessment is displayed in Fig. 2**.**

The Egger’s test was conducted to assess publication bias (supplementary information). No publication bias was observed in Egger’s tests for technical success (*p* = 0.734), clinical success (*p* = 0.727), and IPSS (*p* = 0.476), except than for oncological efficacy (*p* = 0.014)*.*

In addition, sensitivity analysis was performed to assess the influence of each individual study on the overall meta-analysis summary estimate for technical success, clinical success, oncological efficacy and IPSS. The estimates after omission of each individual study were similar to the overall estimate for each outcome. Therefore, no study had a significant impact on the meta-analysis results how to consider removing it. The tables and plots are in the supplementary information.

### Effectiveness Outcomes

**Fig. 3:**
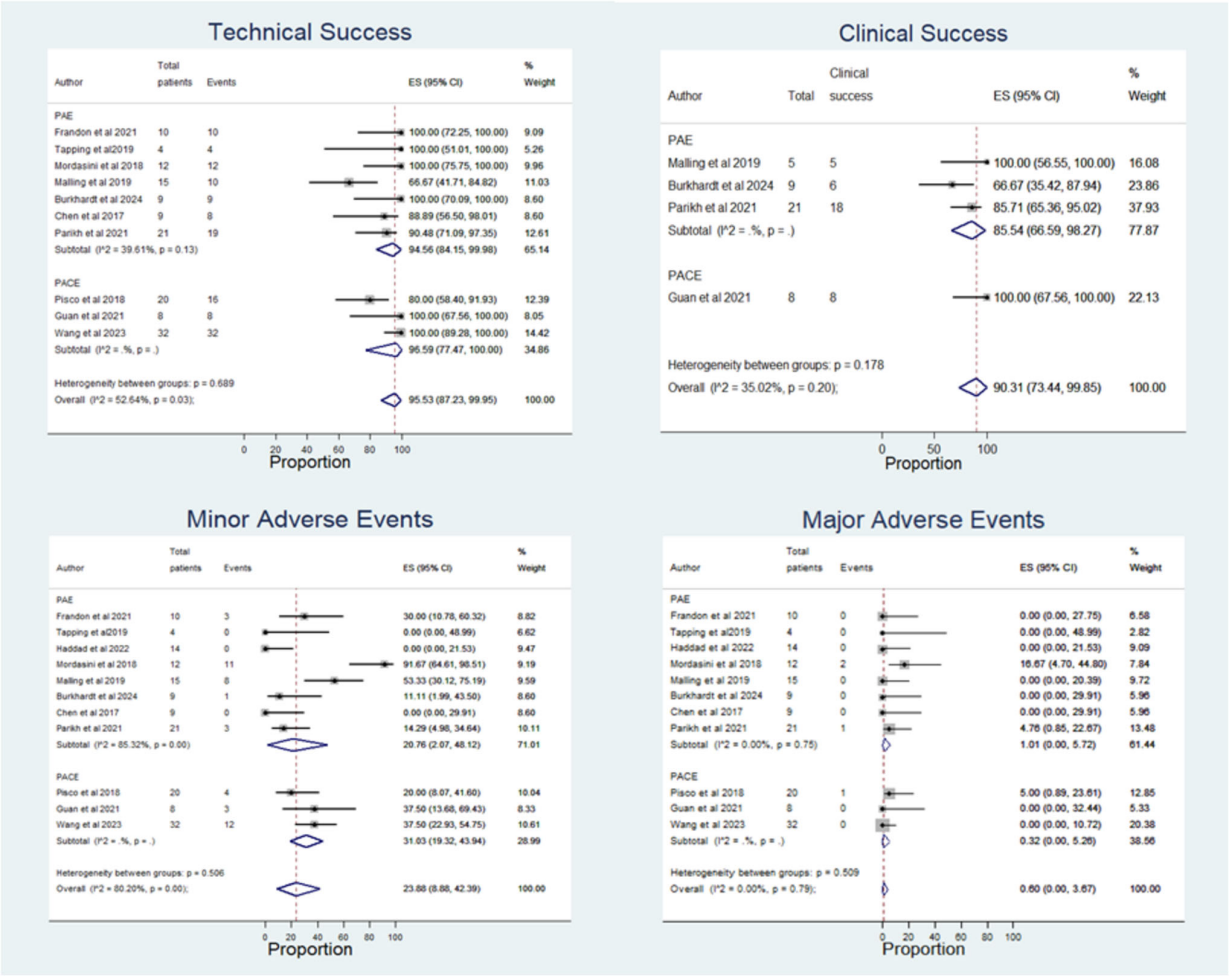
Forest plots of primary outcomes in patients with PCa after either PAE or PACE treatment

**Fig. 4:**
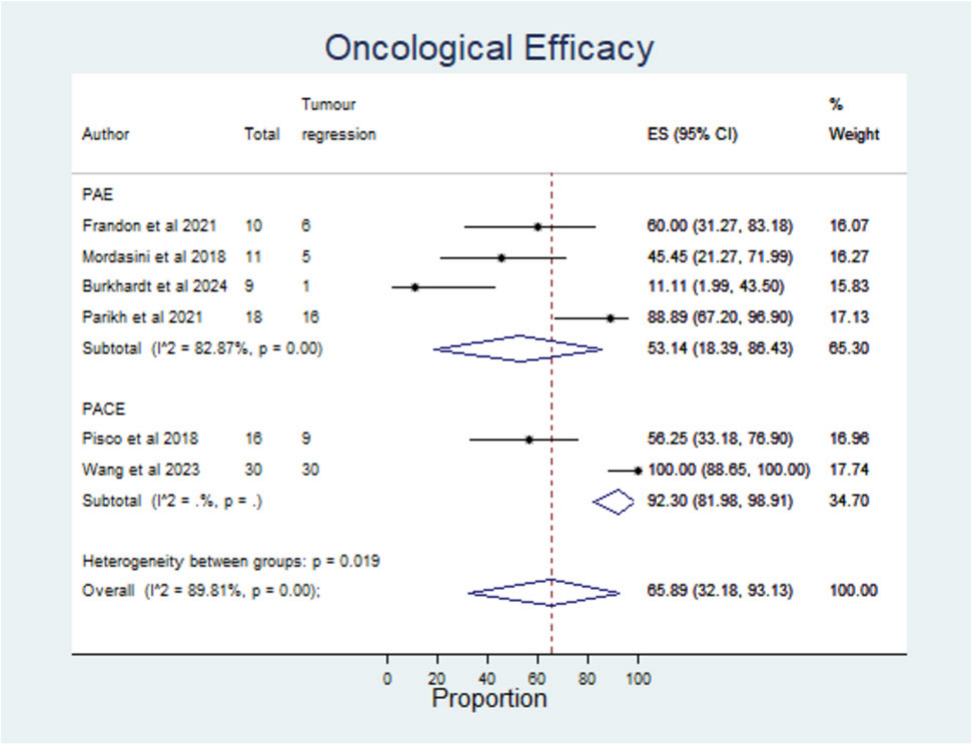
Forest plot of oncological efficacy of either PAE or PACE in patients with PCa

The technical success was reported in 10 out of 11 studies [7–9, 11, 15–20] (Fig. 3), with an overall rate of 95.53% (95% CI: 87.23, 99.95). The clinical success was only reported in 4 out of 11 studies [7–9, 20] (Fig. 3). Overall, 90.31% (95%CI: 73.44, 99.85) of patients achieved a reduction of IPSS at least 30% after treatment. Regarding oncological efficacy, 6 out of 11 studies [8, 9, 11, 15, 18, 19] provided data on PCa tumour response after treatment by MRI (Fig. 4**, **Table 2). The overall proportion of patients with oncological efficacy was 65.89% (95%CI: 32.18, 93.13).

**Table 2:** Oncological outcomes after either PAE or PACE

Study	PSA (ng/ml)	MRI findings	Histology/Survival
Frandon et al. (2021)	Baseline: 6.22 ± 2.23 At 6 months after PAE: 4.4 ± 2.37	• Complete response on mpMRI (no visible PIRADS v2 lesion) in 3 patients • The size of the target lesion on mpMRI was stable at 10 mm (7–16) • Ischaemia was visible at 2 weeks after PAE, not at 6 months • Prostatic ischaemia was partial and heterogeneous, affecting 20% (10–40) of tissue	• Four patients (40%) reported both negative targeted and systematic biopsies • One patient (10%) had PCa progression on a systematic biopsy at 6 months → underwent to EBRT at 1 year Nine patients (90%) were still under active surveillance at 1 year
Tapping et al. (2019)	NA	NA	NA
Haddad et al. (2022)	Baseline: 5.9 ± 2.9 At 2 months after PAE: NA	NA	NA
Mordasini et al. (2018)	Baseline: 5.72 (2.81–16.7) At 6 weeks after PAE: 1.84 (0.08–9.61)	Correlation of MR imaging TRG with histological exam in 8 out of 12 patients (66.7%) Overestimated tumour regression on MRI in 3 patients	Prostate specimens from RARP after PAE: Areas of fibrosis, necrosis, and inflammation were observed in all specimens Ischaemia was more pronounced in the central gland TRG: • Complete response (absence of residual cancer cells and extensive fibrosis of the tumour bed): 16.7% (n = 2) • Strong response (predominant fibrosis of the tumour bed and residual cancer cells occupying < 50% of this area): 0% (n = 0) • Weak response (predominant residual cancer cells exceeding tumour bed fibrosis, ≥ 50% of this area occupied by cancer cells): 41.7% (n = 5) • No response (absence of regressive changes): 41.7% (n = 5)
Malling et al. (2019)	Baseline: 20.3 ± 20.1 Changes after PAE: • At 1 month: 0.78 (95%CI: -3.30, 4.87) • At 6 months: 16.42 (95%CI: -9.89, 42.73)	NA	NA
Burkhardt et al. (2024)	Baseline: 12.6 (0.75–602) After PAE: • At 6 weeks: 12.6 (0.62–839) • At 3 months: 12.6 (0.62–839) • At 12 months: 1.57 (0.66–47.5)	Tumour volume increased slightly from a median of 6.4 (4.6–18.3) to 8.1 (2.4–25.6) ml at 3 months after PAE Viable tumour (no tumour regression on MRI): • > 50% in 8 patients (baseline PV < 30 ml) • 10–50% in one patient (baseline PV < 110 ml)	NA
Pisco et al. (2018)	Baseline: 6.87 ± 6.66 After PACE: • At 1 month: 2.30 ± 3.19 • At 18 months: 1.20 ± 0.96 BS (PSA < 2 ng/ml after PACE): • Short-term: (1–12 months): 75% (12/16) • Mid-term (12–18 months): 62.5% (10/16) BF (PSA > 2 ng/ml after PACE): 3 cases BR (PSA > 2 ng/ml after BS): 3 cases	Patients with Gleason score 6 did not show any change before or after PACE (n = 7) Patients with Gleason score ≥ 7 showed a > 50% reduction in tumour size and ischaemia (3/9)	NA
Chen et al. (2017)	NA	NA	NA
Guan et al. (2022)	Baseline: 326.3 ± 418.8 After PACE: • At 1 week: 361.89 ± 445.61 • At 1 month: 114.14 ± 167.48 • At 3 months: 56.20 ± 104.61	NA	NA
Wang et al. (2023)	Baseline: 48.5 ± 35 After PACE: • At 1 month: 2.30 ± 3.19 • At 18 months: 1.20 ± 0.96 BS (PSA < 2 ng/ml after PACE): • At 1 month: 86.37% (26/30) • At 6–24 months: 73.3% (22/30) BR (PSA > 2 ng/ml after BS): 13.3% (4/30)	The mean prostatic ischaemia was 90% (80–100%) at 1 month after PACE Objective response was 100%: • Complete response in 22 patients (73.3%) • Partial response in 8 patients (26.6%)	Survival rate after PACE: • At 1 year: 96.7% • At 2 years: 86.7% • At 3 years: 73.35%
Parikh et al. (2021)	Baseline: 8.64 (0.43–27.63) Change after PAE: • At 6 weeks: -5.6 (-3 -25.2) • At 3 months: -6.5 (-0.3 -24.5)	No oncologic progression at 6 and 12 weeks: • No new PIRADS 4 lesions • No baseline PIRADS 4 increased to PIRADS 5	NA

BF, Biochemical Failure; BS, Biochemical Success; BR, Biochemical Recurrence; EBRT, External Beam Radiotherapy; mpMRI, Multiparametric Magnetic Resonance Imaging; PAE, Prostatic Artery Embolization; PACE, Prostatic Artery Chemoembolization; PIRADS, Prostate Imaging Reporting and Data System; PSA, Prostate-Specific Antigen; RARP, Robotic-Assisted Radical Prostatectomy; TRG, Tumour Regression Grade

**Fig. 5:**
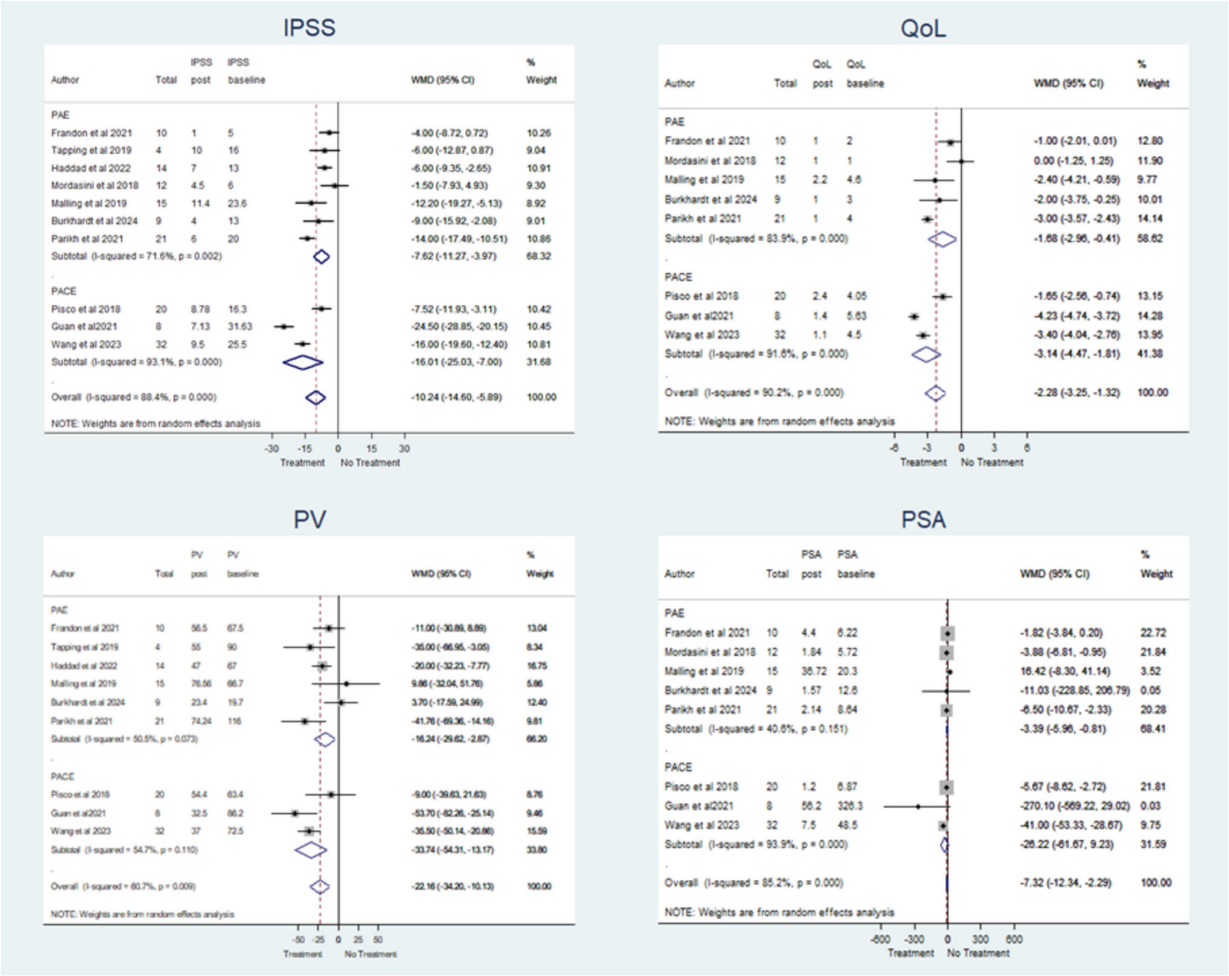
Forest plots of secondary outcomes in patients with PCa after either PAE or PACE

Pairwise meta-analysis of subjective urological outcomes (IPSS and QoL) regarding baseline data was also conducted (Fig. 5**, **Table 3). Statistically significant improvements (p = 0.000) were found after treatment with a mean reduction of 10.24 (95%CI: -14.60, -5.89) for IPSS and 2.28 (95%CI: -3.25, -1.32) for QoL. In addition, there was a significant decrease in PV of 22.16 (95%CI: − 34.20, − 10.13; *p* = 0.009) and PSA of 7.32 (95%CI: − 12.34, − 2.29; *p* = 0.000) (Fig. 5).

**Table 3:** Urological outcomes after either PAE or PACE

Study	IPSS	QoL	Qmax (ml/s)	PVR (ml)	PV (ml)
Frandon et al. (2021)	Baseline: 5 (1–16) After PAE: • At 1 month: 2 (1–6) • At 3 months: 2 (1–9) • At 6 months: 1 (1–19)	Baseline: 2 (0–4) After PAE: • At 1 month: 1 (0–4) • At 3 months: 1 (0–3) • At 6 months: 1 (0–3)	NA	NA	Baseline: 67.5 ± 26 After PAE: • At 2 weeks: 64.4 ± 26.39 • At 6 months 56.5 ± 18.51
Tapping et al. (2019)	Baseline: 16 (8–20) After PAE: • At 3 months: 12 (7–17) • At 12–18 months: 10 (4–12)	EQ-5D-5L: • At baseline: 50 • At 12–18 months: 85	NA	NA	Baseline: 90 (60–120) After PAE: • At 3 months: 60 (48–90) • At 12–18 months: 55 (45–75)
Haddad et al. (2022)	Baseline: 13 ± 5 At 2 months after PAE: 7 ± 4	NA	NA	NA	Baseline: 67 ± 17 At 2 months after PAE: 47 ± 16
Mordasini et al. (2018)	Baseline: 6 (0–23) At 6 weeks after PAE: 4.5 (0–29)	Baseline: 1 (0–4) At 6 weeks after PAE: 1 (0–6)	Baseline: 12.1 (4.9–23.9) At 6 weeks after PAE: NA	Baseline: 5 (0–100) At 6 weeks after PAE: NA	NA
Malling et al. (2019)	Baseline: 23.6 ± 5.3 Changes after PAE: • At 1 month: -9.6 (95%CI: -18.32, -0.88) • At 6 months: -12.2 (95%CI: -23.53, -0.87)	Baseline: 4.6 ± 1.1 Changes after PAE: • At 1 month: -2.4 (95%CI: -4.48, -0.32) • At 6 months: -2.4 (95%CI: -5.39, 0.59)	Baseline: 11.0 ± 7.1 After PAE: NA	Baseline: 171.6 ± 128.1 After PAE: NA	Baseline: 66.7 ± 42 Changes after PAE: • At 1 month: 1.01 (95%CI: -14.73, 16.76) • At 6 months: 9.86 (95%CI: -32.30, 52.03)
Burkhardt et al. (2024)	Baseline: 13 (8–26) After PAE: • At 3 months: 7 (4–26) • At 12 months: 4 (1–10)	Baseline: 3 (1–6) After PAE: • At 3 months: 4 (0–6) • At 12 months: 1 (1–12)	Baseline: 8.9 (0–24.9) After PAE: • At 3 months: 11.1 (0–16.4) • At 12 months: 15.8 (3.2–19.6)	Baseline: 70 (20–600) After PAE: • At 3 months: 10 (0–280) • At 12 months: 26 (0–80))	Baseline: 19.7 (6.4–110.8) After PAE: • At 3 months: 23.4 (2.4–66.3) • At 12 months: NA
Pisco et al. (2018)	Baseline: 16.3 ± 9.0 At 18 months after PACE: 8.78 ± 4.49	Baseline: 4.05 ± 1.64 At 18 months after PACE: 2.4 ± 1.26	Baseline: 15.9 ± 8.3 At 18 months after PACE: 26.1 ± 27.8	Baseline: 15.9 ± 8.3 At 18 months after PACE: 83.9 ± 77.5	Baseline. 63.4 ± 9.0 At 18 months after PACE: 54.4 ± 40.6
Chen et al. (2017)	NA	NA	NA	NA	NA
Guan et al. (2022)	Baseline: 31.63 ± 5.66 After PACE: • At 1 week: 21.5 ± 8.44 • At 1 month: 10.50 ± 3.70 • At 3 months: 7.13 ± 2.70	Baseline: 5.63 ± 0.52 After PACE: • At 1 week: 4 ± 1.31 • At 1 month: 1.86 ± 0.64 • At 3 months: 1.38 ± 0.52	NA	NA	Baseline: 86.20 ± 37.70 After PACE: • At 1 week: 73.81 ± 34.86 • At 1 month: 40.58 ± 17.89 • At 3 months: 32.50 ± 16.66
Wang et al. (2023)	Baseline: 25.5 ± 8.5 After PACE: • At 1 month: 14.5 ± 10.5 • At 12 months: 9.0 ± 5.0 • At 36 months: 9.5 ± 6.0	Baseline: 4.5 ± 1.5 After PACE: • At 1 month: 2.0 ± 1.6 • At 12 months: 1.0 ± 0.8 • At 36 months: 1.1 ± 1.1	Baseline: 8.5 ± 3.5 After PACE: • At 1 month: 13.5 ± 5.5 • At 12 months: 17.0 ± 7.0 • At 36 months: 15.0 ± 8.0	Baseline: 120 ± 50 After PACE: • At 1 month: 65 ± 45 • At 12 months: 50 ± 30 • At 36 months: 48 ± 20	Baseline: 72.5 ± 40.5 After PACE: • At 1 month: 64.5 ± 37.0 • At 12 months: 40.4 ± 21.5 • At 36 months: 37.0 ± 12.0
Parikh et al. (2021)	Baseline: 20 (8–29) Change after PAE: • At 6 weeks: -12 (4–23) • At 3 months: -14 (3–25)	Baseline: 4 (2–6) Change after PAE: • At 6 weeks: -2 (1–5) • At 3 months: -3 (1–4)	NA	NA	Baseline: 116 (14–250) Change after PAE: • At 6 weeks: -24% (-2% -69%) • At 3 months: -36% (-18% -59%)

EQ-5D-5L, EuroQuality of life-5 Dimensions-5 Levels; IPSS, International Prostate Symptom Score; PAE, Prostatic Artery Embolization; PACE, Prostatic Artery Chemoembolization; PV, Prostate Volume; PVR, Post-Void Residual Volume; Qmax, Peak Flow Rate; QoL, Quality of Life

### Safety Outcome

The adverse events were reported in all included studies (Fig. 3, Table 4). The overall pooled rate for minor adverse events was 23.88% (95%CI: 8.88, 42.39), and for major adverse events was 0.60% (95%CI: 0.00, 3.67).

**Table 4:** Complication control and adverse events after either PAE or PACE

Study	Complication control	Minor adverse events	Major adverse events
Frandon et al. (2021)	NA	• Urinary infection (n = 1) • Prostate pain (n = 1) • Groin haematoma (n = 1)	0
Tapping et al. (2019)	• Stopping haematuria (100%) at 3 months • Recurrence of haematuria (n = 1) at 13 months after PAE → Successful re-PAE	0	0
Haddad et al. (2022)	NA	0	0
Mordasini et al. (2018)	NA	• Urgency (n = 4) • Incontinence (n = 1) • Pelvic pain (n = 5) • Rectal bleeding (n = 1)	Bladder wall necrosis (n = 2)
Malling et al. (2019)	Removing urinary catheter in 1/6 patients (17%)	• Groin haematuria (n = 1) • Balanitis (n = 1) • Pelvic pain (n = 1) • Urinary tract infection (n = 1) • Post-embolization syndrome (n = 4)	0
Burkhardt et al. (2024)	NA	• Pelvic pain (n = 1)	0
Pisco et al. (2018)	NA	• Sexual dysfunction (n = 2) • Acute urinary retention (n = 1) • Transient urinary urgency (n = 1)	Bladder wall necrosis (n = 1)
Chen et al. (2017)	Stopping haematuria 6/9 patients (67%) at 3 months	0	0
Guan et al. (2022)	• Stopping haematuria in 2 patients (100%) within 1 month • Removing urinary catheter within 1 week (100%)	• Perineal punctate skin defect (n = 1) • Ischaemia in penile urethral orifice (n = 1) • Rectal wall ischaemia (n = 1)	0
Wang et al. (2023)	• Stopping haematuria in 32 patients (100%) within 1 week • Removing urinary catheter in 17/22 patients (77.3%) within 1 month → 2 patients had recurrence with bladder catheterization at 6 and 14 months	• Urethral burning (n = 11) • Frequency (n = 9) • Perineal pain (n = 10) • Rectal bleeding (n = 6)	0
Parikh et al. (2021)	NA	• Bladder spasm (n = 2) • Urinary retention (n = 1)	• Penile necrosis (n = 1)

### Meta-Regression Analysis

Due to the high heterogeneity found in the meta-analysis, a meta-regression was conducted to explore the source of heterogeneity. Baseline covariates, including patient’s age, IPSS, PV, PSA and PCa stage (localized or advanced), were analysed. No baseline factors were associated with effect on the technical success, clinical success, oncological efficacy and IPSS results. The figures and data are displayed in the supplementary information.

## Discussion

At the present, the treatment options for patients with PCa are extensive and vary depending on the stage of disease. For localized PCa, radical prostatectomy and radiotherapy are the standard treatments, whereas the androgen deprivation therapy alone or in combination with other remains essential in the management of advanced PCa [1]. However, radical prostatectomy carries a high risk of urinary incontinence and erectile dysfunction [21], while radiotherapy can cause acute and late gastrointestinal and urinary toxicities [22]. The primary challenge of oncological treatment is to achieve optimal local tumour control with minimal complications. Accordingly, focal therapies have emerged as promising and minimally invasive strategies, being PAE investigated for PCa treatment due to its minimally invasive ablative nature and fewer adverse events [23].

This meta-analysis included eleven single-arm studies involving patients with PCa treated with either PAE or PACE. The results indicated that both techniques are feasible, effective, and safe for treating LUTS in patients with concurrent PCa, with an overall pooled proportion of 95.53% and 90.31% for technical and clinical successes, and 23.88% and 0.6% for minor and major adverse events, respectively.

In the early stages of PCa, the disease is asymptomatic. As the disease progresses, prostate tumour can induce LUTS, and in more severe cases haematuria and urinary retention. The incidence of LUTS is higher in advanced PCa, with approximately half of the patients developing these symptoms. In this meta-analysis, clinical success was defined as the relief of LUTS at least 30% according to primary endpoint for PAE effectiveness in BPH [24]. PAE achieved a clinical success rate of 90.31%, reducing significantly IPSS (-10.24 points) and QoL (-2.28 points). In addition, either PAE or PACE has also demonstrated its efficacy to treat several complications, such as gross haematuria and urinary retention [5, 6, 25, 26]. Therefore, this meta-analysis has demonstrated that either PAE or PACE also improves the subjective urological outcomes, as well as controlling several complications in patients with PCa.

The underlying mechanism of the therapeutic effect of PAE is to induce a prostate ischaemia and, consequently, prostate shrinkage, even in large prostate (≥ 100 mL) [27, 28]. The results have shown a statistically significant decrease in mean PV along with a decrease of PSA after treatment. Haddad et al. [10] used PAE as neoadjuvant treatment in 13 patients with large PV(> 60 mL) and moderate-to-severe LUTS with the purpose of reducing these contraindications for permanent interstitial brachytherapy. The results showed a significant PV reduction (< 60 mL) in 86% of cases, which enabled the application of the local therapy. In addition, other studies have also assessed the benefits of PAE as an adjuvant treatment to external beam radiotherapy for treating LUTS secondary to an enlarged prostate in patients with concomitant PCa [8, 29, 30]. In this context, PAE was an effective adjuvant treatment, providing a relief of LUTS and a reduction of PV, which led to a reduction in radiation doses and radiotherapy-related toxicity in patients with PCa.

Multiparametric MRI has a high sensitivity (84%) and specificity (39%) in the detection of clinically significant PCa (Gleason score ≥ 7), and the lesion correlates with histological findings. However, MRI is less sensitive in cases of low-risk PCa, and an overestimation of tumour regression may be reported [31]. Despite the relatively good oncological results observed on MRI in this meta-analysis (65.59%), this result could be subject to bias, since both PAE and PACE failed to cure PCa, achieving a complete response with absence of PCa cells in the histological exam in only 16.7% and 20% of patients with PCa [11, 15]. Although PAE has shown not to be a curative treatment, it may be considered as an option that does not preclude subsequent curative therapies, such as surgery or radiation at a later date [32].

The use of transarterial chemoembolization (TACE) has been extended from hepatocellular carcinoma (HCC) to other vascular tumours as an alternative treatment for those cases refractory or contraindicated to other treatments, offering oncological efficacy and safety [33]. TACE improves the overall survival in patients with HCC, but the benefit of adding a chemotherapeutic agent in TACE remains controversial. TACE has not been superior to bland embolization in terms of survival and tumour response, demonstrating that bland embolization can be as effective as TACE, even with fewer adverse events [34] Although PACE has shown better results regarding oncological efficacy and urological outcomes, these results should be interpreted with caution because only three out of eleven included studies used PACE for the treatment of PCa, employing different drugs, embolic agents, and delivery approaches, which may introduce bias into the results [18–20].

The minimally invasive nature of PAE with few complications related to the procedure [35, 36] has been also confirmed in this meta-analysis, with low rates of minor and major adverse events in patients with PCa. The results confirm the safe profile of PAE and PACE to treat urological symptoms in patients with PCa, although PAE has been associated with lower rates of minor (20.76% vs 31.03%) and major (1.01% vs 0.32%) complications than PACE, similar to another meta-analysis where the bland embolization reported similar or less adverse effects than TACE [34]. However, these results could be biased due to the variability between studies.

The limitations of this meta-analysis include the lack of randomized controlled trials with standard treatments and the absence of studies with longer follow-ups. Another limitation was the inadequate description of data for meta-analysis, with range as measure of variation instead of standard deviation (SD). Although SD is not reported, it can be extracted from other metrics, such as changes, standard error, or CI, but range can be more challenging [37]. To address this issue, SD was estimated from ranges by Walter and Yao’ method, using an appropriate conversion factor according to sample size [38]. In addition, the overall heterogeneity was high due to the variability in the baseline characteristics of the included studies, such as the number of patients, PCa stage (localized or advanced PCa), symptoms, embolization technique (PAE vs PACE) and follow-up durations. However, meta-regression analysis was performed in order to identify any factor which might affect to high heterogeneity of this meta-analysis, but no factors were found. The results of this meta-analysis should be interpreted with caution due to the high heterogeneity, small sample size, high risk of bias and the limited number of the included studies.

## Conclusion

The available evidence suggests that PAE and PACE are feasible, effective and safe techniques for the treatment of urological symptoms with few adverse events in patients with concurrent PCa. However, the curative effect of either PAE or PACE has shown to be insufficient as a primary treatment of PCa. Both techniques can effectively manage the local symptoms in patients with PCa, such as LUTS, urinary retention and haematuria, with a low risk of severe adverse events. This offers potential benefits from a palliative perspective or even as an adyuvant treatment with local therapies in patients with localized PCa.

The role of PAE in PCa management remains uncertain, and further research involving larger population and well-designed prospective trials is needed to clarify its benefits. Future studies should focus on patient selection criteria, procedural requirements, follow-up, and other factors, with the aim of establishing prostate embolization as a complementary tool in the management of PCa in patients.

## Electronic supplementary material

Below is the link to the electronic supplementary material.

Supplementary file 1 (DOCX 18.5 KB)
