# Supplementary material for: Effectiveness and Safety of Prostatic Artery Embolization for Patients with Prostate Cancer: A Systematic Review and Meta-Analysis
Source: Cardiovasc Intervent Radiol. 2025 Jul 10;48(9):1263–77. doi: 10.1007/s00270-025-04107-6 (PMC12464020; doi:10.1007/s00270-025-04107-6)

SUPPLEMENTARY INFORMATION

TABLE OF CONTENTS

[SEARCH STRATEGY 2](#_Toc197674309)

[META-REGRESSION 3](#_Toc197674310)

[A. Technical success 3](#_Toc197674311)

[B. Clinical success 6](#_Toc197674312)

[C Oncological efficacy 9](#_Toc197674313)

[D. IPSS after treatment 12](#_Toc197674314)

[EGGER’S TEST 15](#_Toc197674315)

[A. Technical success 15](#_Toc197674316)

[B. Clinical success 16](#_Toc197674317)

[C Oncological efficacy 17](#_Toc197674318)

[D. IPSS after treatment 18](#_Toc197674319)

[SENSITIVITY ANALYSIS 19](#_Toc197674320)

[A. Technical success 19](#_Toc197674321)

[B. Clinical success 20](#_Toc197674322)

[C. Oncological efficacy 21](#_Toc197674323)

[D. IPSS after treatment 22](#_Toc197674324)

# SEARCH STRATEGY

1. PUBMED

(((((((((prostat* artery embolization[Title/Abstract]) OR (prostat* artery embolisation[Title/Abstract])) OR (PAE[Title/Abstract])) OR (prostat* artery chemoembolization[Title/Abstract])) OR (prostat* artery chemoembolisation[Title/Abstract])) OR (transarterial chemoembolization[Title/Abstract])) OR (transarterial chemoembolisation[Title/Abstract])) OR (TACE[Title/Abstract])) OR (PACE[Title/Abstract])) AND (prostate cancer[Title/Abstract])

1. WEB OF SCIENCE/SCOPUS

(TS=("prostat* artery embolization" OR "prostat* artery embolisation" OR "prostat* artery chemobolization" OR "prostat* artery chemobolisation" OR "transarterial chemoembolization" OR "PAE" OR "PACE" OR "TACE")) AND TS=(prostate cancer)

# META-REGRESSION

## Technical success

**
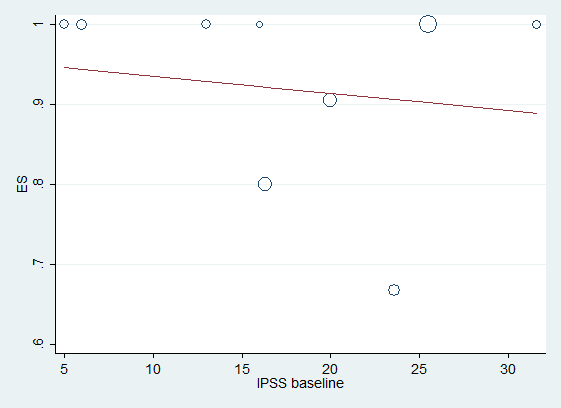
**

**
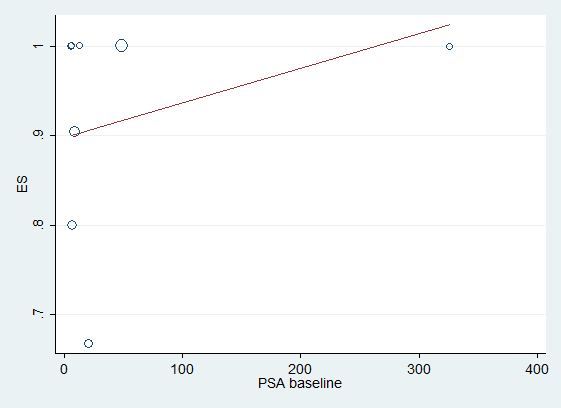

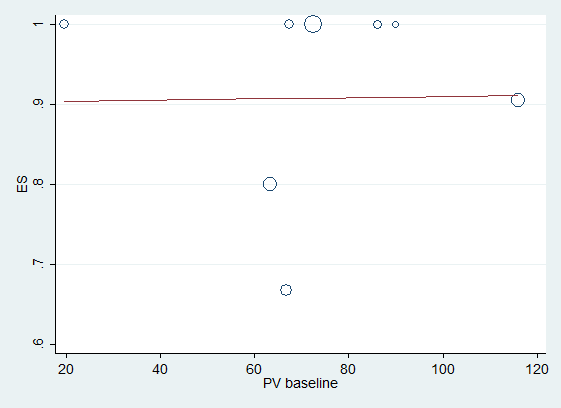
**

**
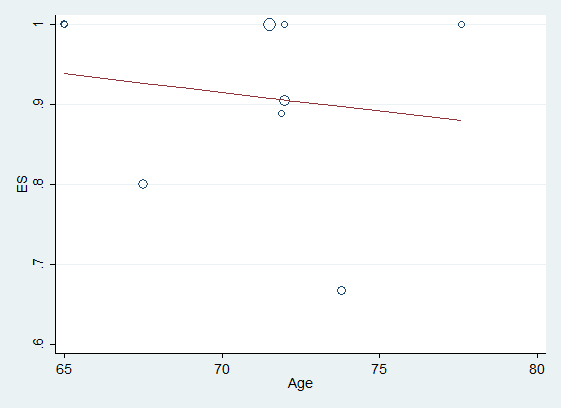

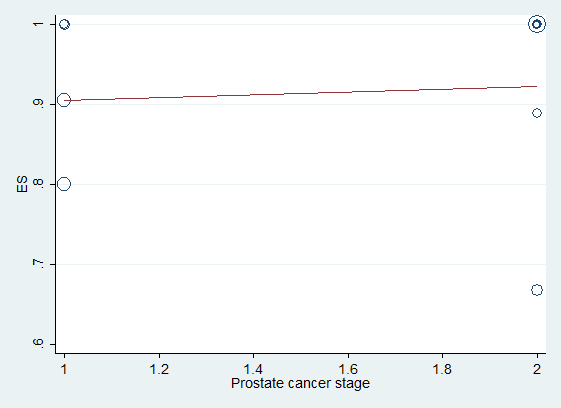
**

1. **Clinical success**

**
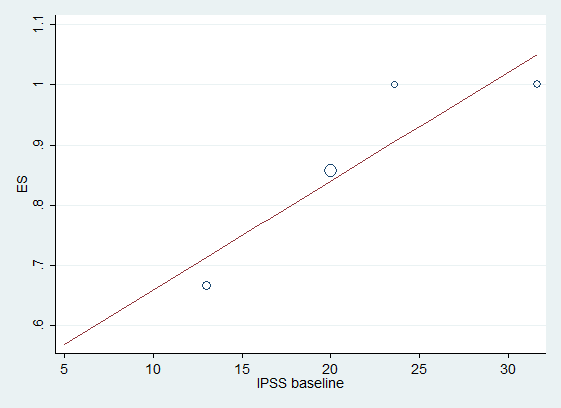
**

**
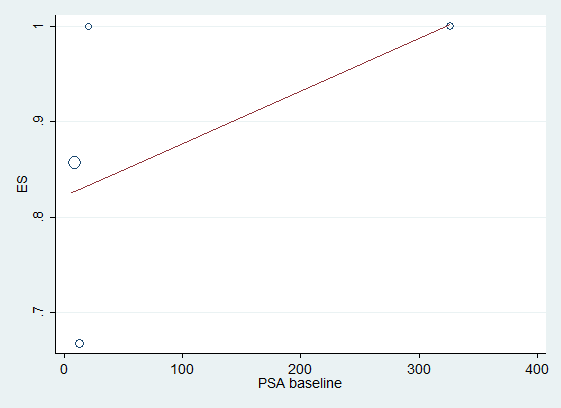

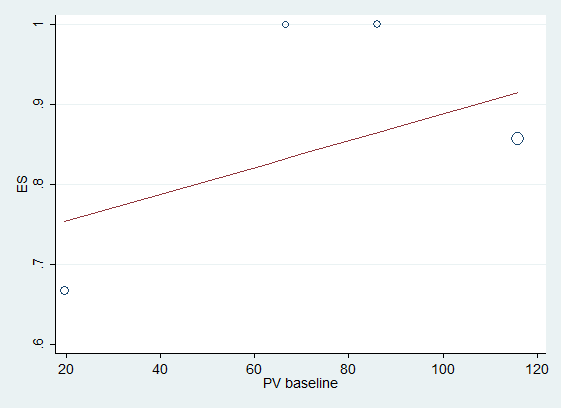

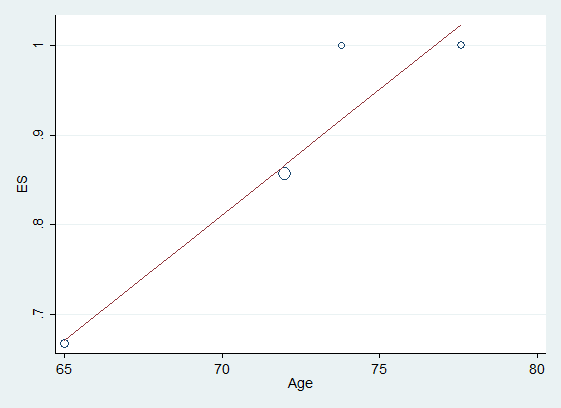

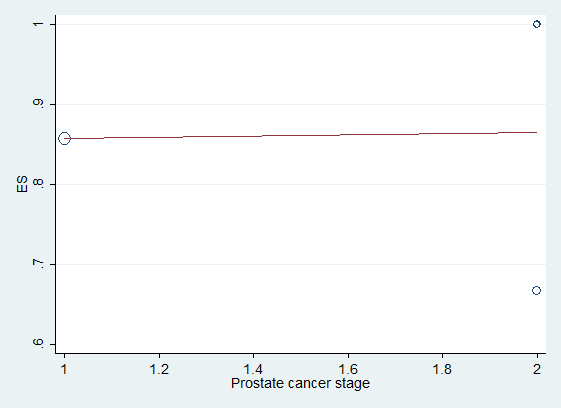
**

## C Oncological efficacy

**
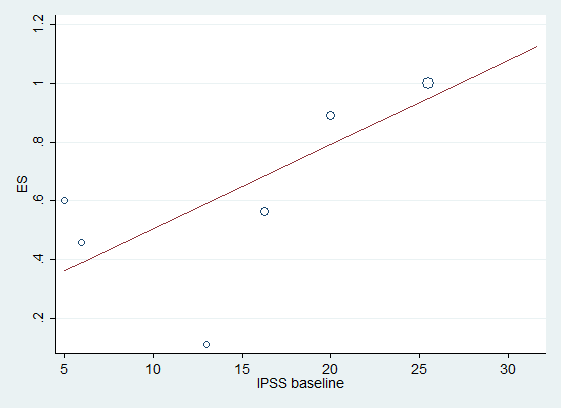
**

**
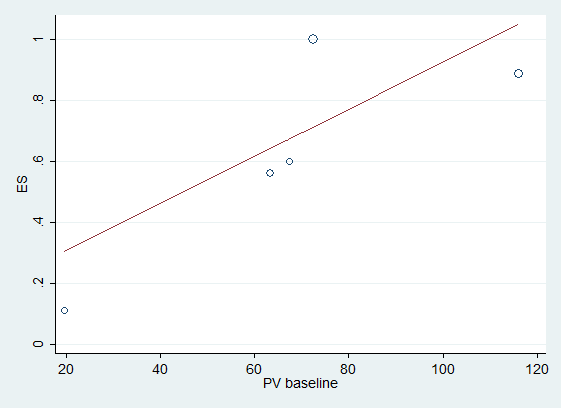

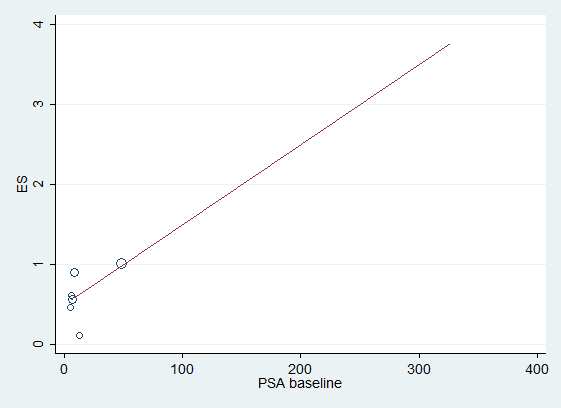
**

**
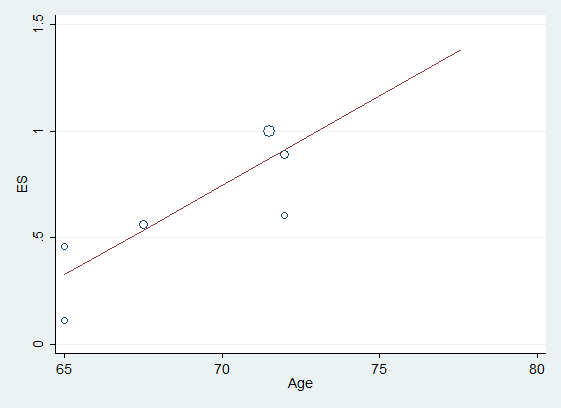

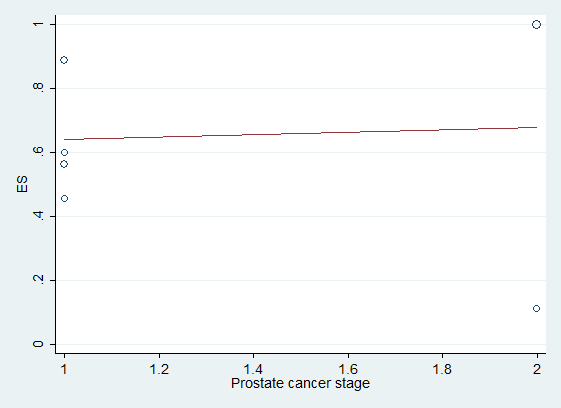
**

## IPSS after treatment

**
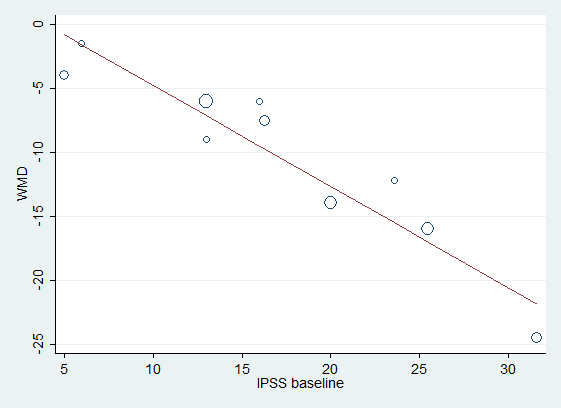
**

**
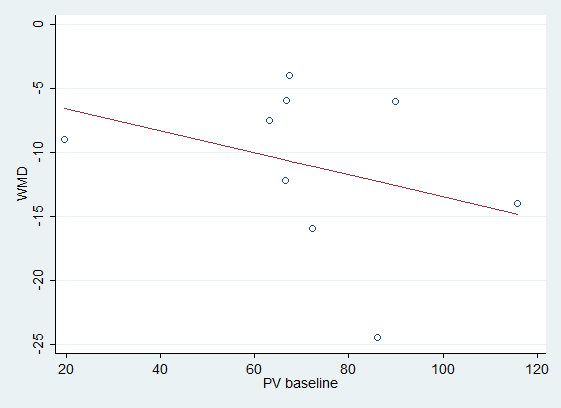

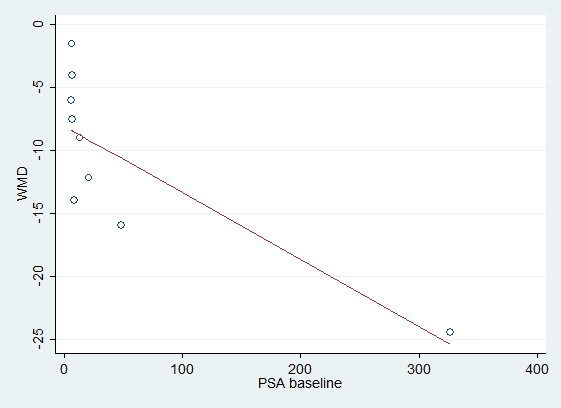

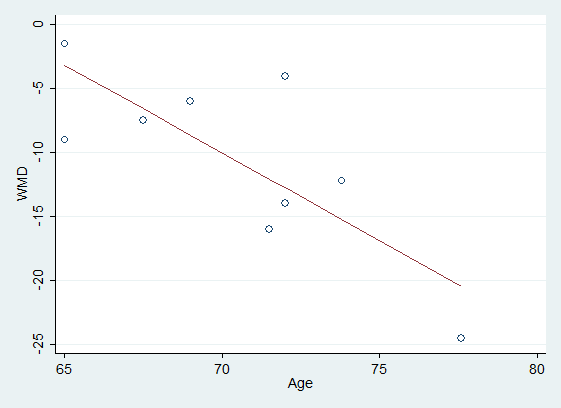

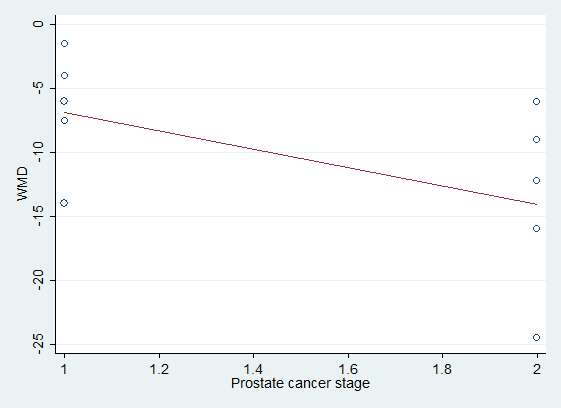
**

# EGGER’S TEST

## Technical success

**
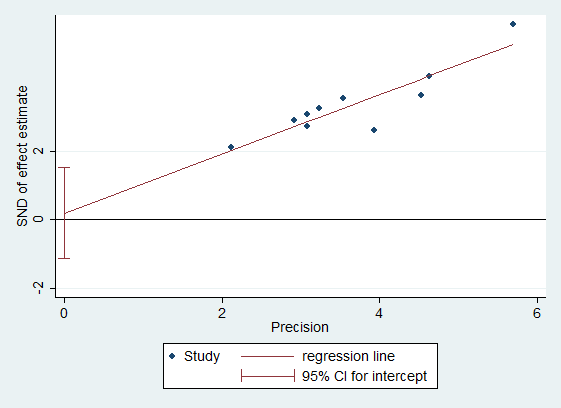
**

1. **Clinical success**

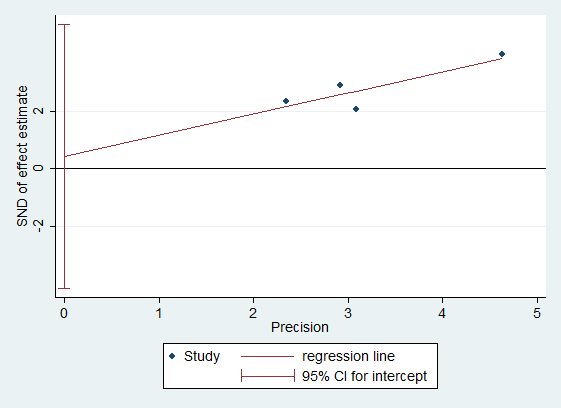


## C Oncological efficacy

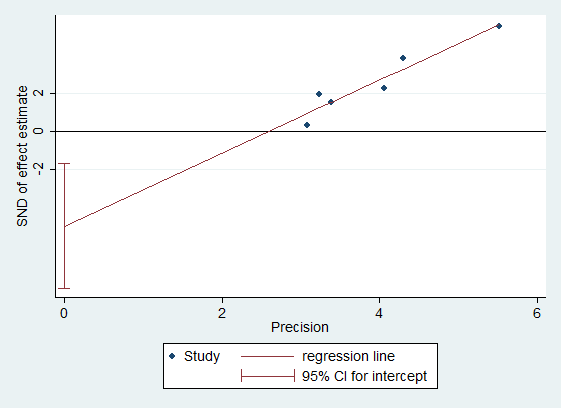


## IPSS after treatment

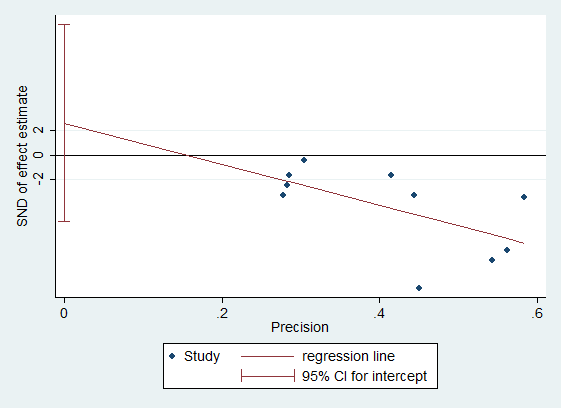


# SENSITIVITY ANALYSIS

1. **Technical success**


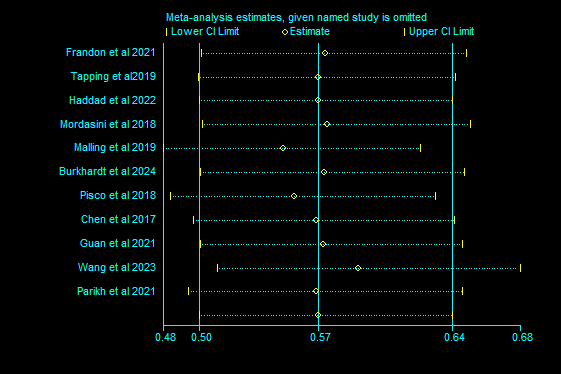


1. **Clinical success**

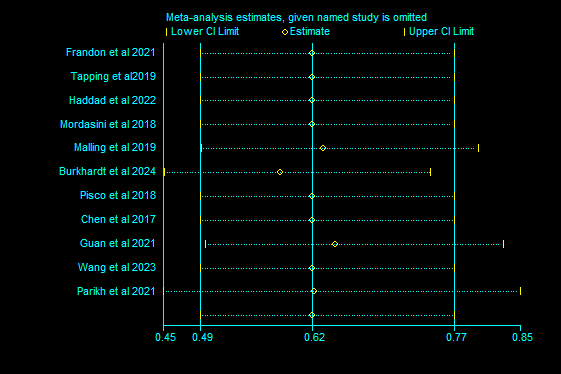


1. **Oncological efficacy**


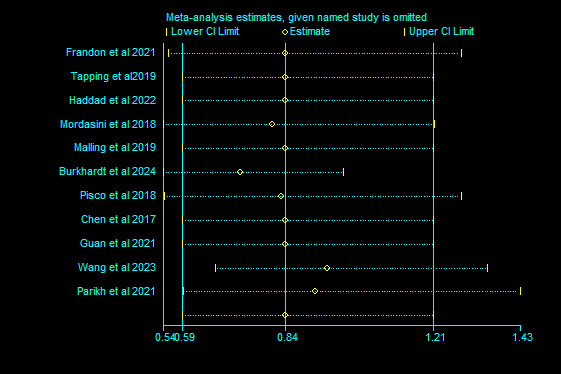


1. **IPSS after treatment**


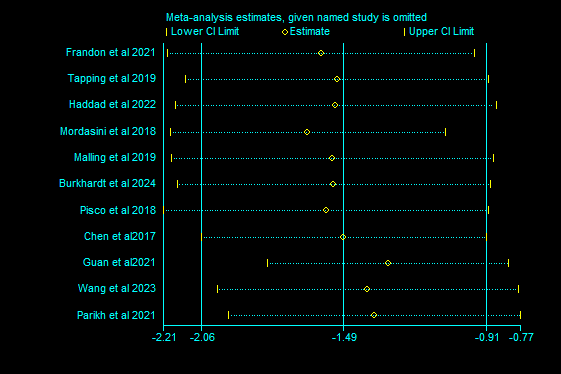

Supplement: Supplementary file 1 — (DOCX 18.5 KB) [file 270_2025_4107_MOESM1_ESM.docx]
